# Amphibians and reptiles of Samar Island Natural Park, Philippines, with an updated checklist, a rediscovery, and new records for Samar Island

**DOI:** 10.3897/zookeys.1269.173854

**Published:** 2026-02-17

**Authors:** Mae Lowe L. Diesmos, Yñigo Luis C. del Prado, Paulo Miguel M. Kim, Niño Andree Louis E. Caguimbal, Russell Evan L. Venturina, Antonio N. Lorenzo II, Arvin C. Diesmos

**Affiliations:** 1 Department of Biological Sciences, University of Santo Tomas, Manila, Philippines Department of Biological Sciences, University of Santo Tomas Manila Philippines; 2 Research Center for the Natural and Applied Sciences, University of Santo Tomas, Manila, Philippines Research Center for the Natural and Applied Sciences, University of Santo Tomas Manila Philippines; 3 The Graduate School, University of Santo Tomas, Manila, Philippines The Graduate School, University of Santo Tomas Manila Philippines; 4 Biodiversity Knowledge Management Department, ASEAN Centre for Biodiversity, Laguna, Philippines Biodiversity Knowledge Management Department, ASEAN Centre for Biodiversity Laguna Philippines

**Keywords:** Biodiversity, endemic, herpetofauna, species list, Mindanao PAIC

## Abstract

The Philippine archipelago is renowned for its exceptional biodiversity and high endemism, particularly among amphibians and reptiles. Samar Island, the third largest in the country and a key element of the Greater Mindanao Pleistocene Aggregate Island Complex, still harbors swathes of old-growth forest and limestone karst systems, making it a critical site for understanding biogeographic and evolutionary patterns in the Philippines. The herpetofaunal diversity of Samar remains poorly documented, with data scattered and woefully outdated. Early collections date back to the mid-19^th^ century, and while recent efforts have incrementally improved species inventories, a comprehensive and updated assessment remains lacking. Here, we present the results of recent intensive surveys conducted within the Samar Island Natural Park (SINP) and adjacent protected areas. Our study reports 79 species from SINP and a total of 104 species from Samar Island, including three new island records, and confirms the continued presence of historically documented taxa. The rediscovery of *Ramphotyphlops marxi* is reported and the most updated checklist of the island’s amphibian and reptile species provided. These findings highlight the importance of sustained biodiversity assessments across the Philippine archipelago to inform conservation strategies.

## Introduction

The megadiverse Philippine archipelago exhibits exceptional species richness and endemism, particularly of its amphibian and reptile faunas (Inger 1954; Brown and Alcala 1970; Mallari et al. 2001; Brown and Diesmos 2009; Diesmos et al. 2014; Heaney et al. 2016). This remarkable diversity is largely shaped by the country’s intricate geological history and its formation as a series of Pleistocene Aggregate Island Complexes (PAICs). Samar Island, the third largest landmass in the country, forms the northernmost extent of the Greater Mindanao Biogeographic Region (Heaney 1985) or the Mindanao Pleistocene Aggregate Island Complex (Brown and Diesmos 2001), which also includes the islands of Leyte, Bohol, and Mindanao. This biogeographical context, coupled with Samar’s extensive tracts of old-growth rainforests and limestone karst formations (Restificar et al. 2006; Patindol 2016), makes it a critical region for understanding Philippine biodiversity.

Despite its ecological significance, a comprehensive understanding of Samar Island’s herpetofauna has remained elusive. Our current knowledge is fragmented, derived from disparate sources spanning nearly two centuries. Early naturalists, such as Dr. Fedor Jagor and Dr. Otto Franz von Möllendorff, collected the earliest specimens of amphibians and reptiles from Samar in the 1850s, leading to descriptions of Philippine endemics like *Pinoyscincus llanosi* (Taylor, 1919) and *Malayotyphlops ruber* (Boettger, 1897). However, comprehensive assessments of the herpetofauna remained scarce. Inger’s 1954 review of Philippine amphibians, for instance, listed only nine species from Samar. Gaulke (1994) listed a total of 72 species and more recent localized surveys (Patindol 2016; Obeña et al. 2021; Villanueva et al. 2021) have confirmed some of these previous records. A taxonomically updated checklist of the herpetofauna for the entire island has been lacking until now.

Recognizing the island’s immense biological value, the Samar Island Natural Park (SINP) was established in 2003 to safeguard its unique ecosystems. Building upon previous efforts (Gaulke 1994; Siler et al. 2009; Patindol 2016; Obeña et al. 2021; Villanueva et al. 2021), we present results of the most recent and intensive herpetofaunal surveys that we conducted within SINP and a comprehensive and updated checklist of the amphibians and reptiles of Samar Island. We highlight notable records, including the first documented occurrence of three species on Samar and the rediscovery of the forest blind snake, *Ramphotyphlops marxi* (Wallach, 1993).

## Materials and methods

### Study area

Samar Island lies southeast of the island of Luzon and northeast of Leyte. It is the third largest island in the Philippines with an entire area of ca 1,310,700 ha (Patindol 2016; Buot et al. 2022). Within the Mindanao PAIC, Samar Island represents the northernmost part of the faunal region, separated only by the San Bernardino Strait from the Luzon PAIC. Situated at its center is the Samar Island Natural Park (SINP; Province of Samar, Eastern Visayas, Philippines), a protected area (Proclamation No. 442) of lowland forests covering 333,300 ha (Buot et al. 2022). SINP is recognized as a Key Biodiversity Area (KBA; Conservation International Philippines et al. 2006) and was recently nominated as a UNESCO World Heritage Site.

SINP ranges from sea level to 811 m in elevation and its forest ecosystem is classified into lowland evergreen rainforest, forest over limestone, and forest over ultrabasic rock. The lowland evergreen rainforest is composed of more than 50% closed canopy and primary lowland dipterocarp forest. The buffer zone is dominated by mixed vegetation composed of coconuts, saplings, brush, shrubs, and grass (65,553 ha or 54% of the buffer zone), followed by natural forest and agricultural land and grasslands. Wetlands, built-up areas, and roads cover ca 785 ha of the buffer zone (UNDP 2007).

Samar Island is characterized by two climate types, the eastern portion has a Type II climate with no dry season and a pronounced maximum rain period from December to February while the western part falls under the Type IV climate where rainfall is roughly evenly distributed throughout the year (PAGASA 2014).

**Figure 1.**
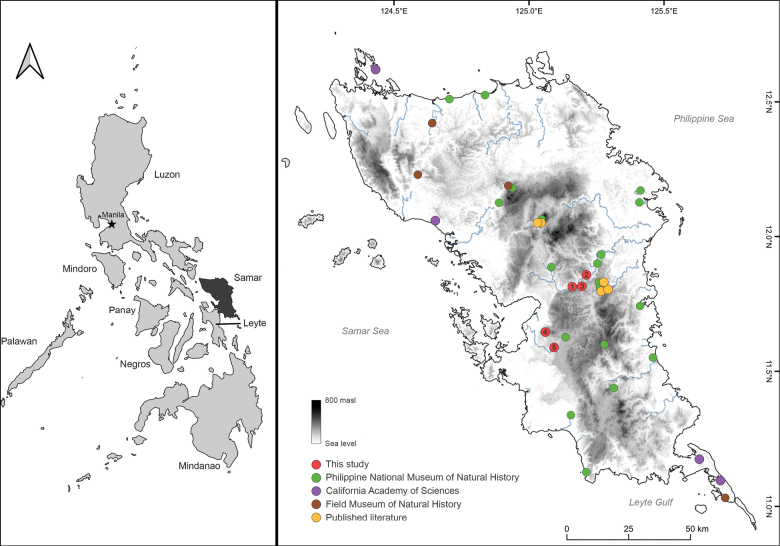
Map of Samar Island showing field surveys and collection sites.

**Figure 2.**
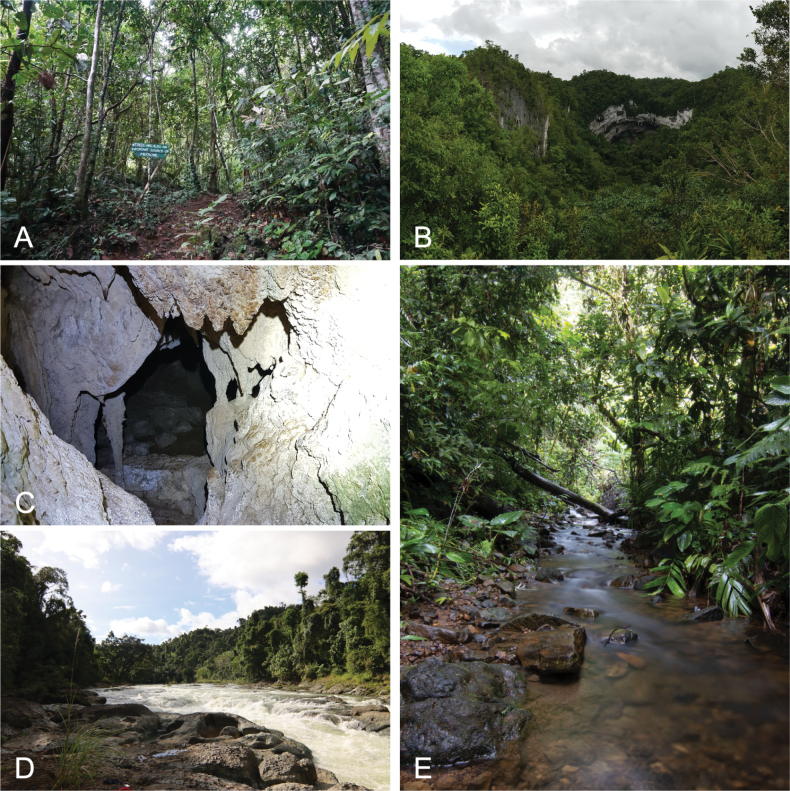
Examples of natural habitat types in Samar Island Natural Park. **A, B**. Forest over limestone; **C**. Cave system; **D, E**. Lowland riparian forest.

Our field studies were conducted along the extensive limestone karst landscape and inland forests of SINP and other localities (Fig. 1). Our five sampling sites were:

- Site 1. Samar Island, Municipality of Paranas, Barangay Tenani, DENR Samar Island Natural Park; N 11.81405, E 125.16095; 107–336 m elevation; 09–11 March 2016, 23–31 October 2021; a lowland forest over limestone site characterized by a calcareous limestone substrate, karst outcrops, caves, dens, and other karst formations (Fig. 2). The main forest trail is characterized by closed canopy forest with emergent trees of more than 30 m in height; trees progressively become stunted as the elevation increases. The more disturbed forest trail has denser secondary growth and small limestone caves are present. A cultivated patch can be encountered enroute to the birding platform and the hanging bridge at the terminus of the tourist trail.
- Site 2. Samar Island, Municipality of Paranas, Barangay San Isidro, Ulot River; N 11.81405, E 125.16095; 49–117 m elevation; 11–16 March 2016, 02–06 November 2021; a mixed habitat type along the Ulot River. The proximate area to the river comprises a fragment of cultivated grassland, coconut plantations, and creeks adjoined by riparian forests. Ascent into the main trail gradually turns into a stretch of forest over ultrabasic soil. Midway through the transect to the terminus is a gradual shift to lowland evergreen forest. Traces of logged trees and man-made routes can be detected along smaller creeks.
- Site 3. Samar Island, Municipality of Paranas, Barangay San Isidro, Liao Cave; N 11.81821, E 125.19424; 204–244 m elevation; 06–09 November 2021; predominantly a limestone cave system, the adjoining limestone forest is similar in stature and type to that of Site 1. Liao Cave is a subterranean system ~ 500 m away from the highway and is populated by humans. Foliage and canopy become progressively denser the farther from the highway. No clear water systems were detected along the trail, though a spring exists as a water source near the edge of the forest. The entrance is heavily overgrown by secondary growth and forest litter. The interior is composed of limestone formations; the substratum is a layer of ultramafic soil.
- Site 4. Samar Island, Municipality of Calbiga, Barangay Panyuran, Langun-Gobingob Cave Trail; N 11.64513, E 125.05816; 119–141 m elevation; 09–11 November 2021; a massive karst cave complex situated in mixed limestone-ultrabasic forest. Secondary growth extends to the entrance of the cave. The exterior substratum is sandy. The interior is composed of several cave galleries, the largest is more than 5 km, 50 m in height, and 40 m in width. Among the silica-formed limestone structures are massive sinkholes, underground rivers, and a spring situated in the Langun gallery.
- Site 5. Samar Island, Municipality of Calbiga, Barangay Literon, Lulugayan Falls; N 11.58656, E 125.09088; 100–200 m elevation; 30 October–02 November 2021; a large river system made up of smaller tributaries adjacent to riparian forests and brush. This strongly lotic water body is characterized by the falls standing adjacent to human-occupied areas. The riparian forests and brush are typically muddy though some degree of limestone formation can be observed near the water edge.

### Sampling and data collection

Our herpetofaunal surveys were conducted in March 2016 and in October to November 2021. We used standard herpetological field techniques (Heyer et al. 1994; Simmons 2002; Dodd 2016), including time-constrained visual encounter surveys, opportunistic searches, patch sampling, and acoustic surveys. Visual encounter surveys were conducted in existing forest trails, along streams, cave entrances, and forest interiors during the day (0700–1200 h) and at night (1900–2400 h). Each survey involved three to six observers, actively searching for the presence of amphibians and reptiles in microhabitats such as forest litter, rotting logs, rock crevices, epiphytes, tree holes, in between tree buttresses, and streambanks. Survey efforts varied between the two field expeditions but generally ranged between 20 and 30 person-hours per site per visit. Acoustic surveys were conducted during the evening to document advertisement calls of frogs, with calling individuals either recorded or located visually whenever possible (Heyer et al. 1994; Royle and Nichols 2003; Dorcas et al. 2009). Across all sites and expeditions, the total survey effort exceeded 710 person-hours.

All voucher specimens were identified during processing and verified using published reference guides (Brown and Alcala 1978, 1980; Alcala and Brown 1998; Diesmos et al. 2008; Weinell et al. 2019). Specimens and associated natural history data were deposited and permanently stored at the Herpetological Research Collection of the University of Santo Tomas in Manila (Institutional code: UST-HRC).

To produce a comprehensive herpetofaunal species list for Samar Island, information from checklists (Inger 1954; Leviton 1963; Gaulke 1994; Diesmos et al. 2015; Patindol 2016; Sanguila et al. 2016; Leviton et al. 2018; Villanueva et al. 2021; Sy 2023) and taxonomic studies (Boettger 1897; Taylor 1919, 1920, 1922; Brown 1956; Wallach 1993; Lanza 1999; McGuire and Alcala 2000; Siler et al. 2009, 2014; Siler and Brown 2010; Welton et al. 2010, 2012, 2017; Brown et al. 2015a, b; Davis et al. 2015; Barley et al. 2020; Diesmos et al. 2020; Weinell et al. 2020; Blanck et al. 2023) were reviewed. Species records and collection data from scientific repositories (Philippine National Museum of Natural History, California Academy of Sciences, and Field Museum of Natural History) available online were also examined.

The conservation status of species is based on the most recent information from the International Union for the Conservation of Nature (IUCN Red List; https://www.iucnredlist.org).

## Results and discussion

During our surveys, we observed a total of 79 species of reptiles and amphibians belonging to 20 families. Of these, 26 are frogs, 31 are lizards, 21 are snakes, and one species of turtle. Among the recorded species, 53 (68%) are known only from the Philippines where 24 are found only in the Mindanao PAIC, five only on the islands of Samar and Leyte, and three are endemic to Samar Island. Additionally, three species of frogs (from two genera) possibly new to science were also recorded. According to the IUCN (2025), four are Near Threatened while two species are Vulnerable. The results of our surveys, combined with a review of available literature, show a total of 104 species of amphibians and reptiles that are known from Samar Island (Table 1).

**Table 1.** Amphibians and reptiles of Samar Island. Site records are from DENR Samar Island Natural Park (I), Ulot River (II), Liao Cave (III), Langun-Gobingob Cave Trail (IV), Lulugayan Falls (V). Habitat types include riparian forest (RF), forest over limestone (FL), secondary growth forest (SG), agricultural plantation (AP), open areas and scrubby vegetation (OS), and built-up areas (B). An asterisk (*) indicates Philippine endemic species.

Taxa		Site	Habitat Type	IUCN Status	Distribution	Source
AMPHIBIANS						
Bufonidae						
1	*Pelophryne lighti* (Taylor, 1920)* Eastern Mindanao dwarf toad	I, II	RF, FL	LC	Mindanao PAIC	This study; Gaulke 1994
2	*Rhinella marina* (Linnaeus, 1758) Cane toad	I, V	AP, OS, B	LC	Throughout the Philippines (Introduced)	This study; Diesmos et al. 2006
Ceratobatrachidae						
3	*Platymantis bayani* Siler, Alcala, Diesmos, & Brown, 2009* Walter’s limestone frog	I, III, IV	FL	DD	Mindanao PAIC (Samar)	This study; Siler et al. 2020
4	*Platymantis corrugatus* (Duméril, 1853)* Masked wrinkled ground frog	I	FL	LC	Throughout the Philippines (Endemic)	This study; Gaulke 1994
5	*Platymantis guentheri* (Boulenger, 1882)* Günther’s wrinkled tree frog	III	FL	LC	Mindanao PAIC	This study; Diesmos et al. 2015
6	*Platymantis navjoti* Diesmos, Scheffers, Mallari, Siler, & Brown, 2020* Navjot Sodhi’s cloud frog	I	FL	-	Mindanao PAIC (Samar & Leyte)	This study; Diesmos et al. 2020
7	*Platymantis rabori* Brown, Alcala, Diesmos, & Alcala, 1997* Rabor’s horned tree frog	I	FL	LC	Mindanao PAIC	This study; Diesmos et al. 2015
Dicroglossidae						
8	*Fejervarya moodiei* (Taylor, 1920) Brackish frog	-	-	LC	Throughout the Philippines (Non-endemic)	Inger 1954; Gaulke 1994
9	*Fejervarya vittigera* (Wiegmann, 1834)* Luzon grass frog	I	SG	LC	Throughout the Philippines (Endemic)	This study; Gaulke 1994
10	*Limnonectes leitensis* (Boettger, 1893)* Philippine swamp frog	I, V	RF, FL	LC	Throughout the Philippines except Luzon, Mindoro, and Palawan PAICs (Endemic)	This study; Inger 1954
11	*Limnonectes magnus* (Stejneger, 1910)* Mindanao fanged frog	I, II, V	RF, FL	NT	Mindanao PAIC	This study; Diesmos et al. 2015
12	*Occidozyga laevis* (Günther, 1858)* Philippine oriental frog	I	RF	LC	Throughout the Philippines (Endemic)	This study; Inger 1954
Megophryidae						
13	*Leptobrachium lumadorum* Brown, Siler, Diesmos, & Alcala, 2010* Mindanao litter frog	I	FL	LC	Mindanao PAIC	This study
14	*Pelobatrachus stejnegeri* (Taylor, 1920)* Mindanao horned frog	I, II	FL	LC	Mindanao PAIC	This study; Inger 1954
Microhylidae						
15	*Kalophrynus sinensis* Peters, 1967* Philippine sticky frog	I, V	RF, FL	-	Mindanao PAIC	This study; Inger 1954
16	*Kaloula picta* (Duméril & Bibron, 1841)* Philippine painted narrowmouth toad	I	AP, OS	LC	Throughout the Philippines (Endemic)	This study; Diesmos et al. 2015
17	*Oreophryne anulata* (Stejneger, 1908)* Mindanao cross frog	I	AP, SG	LC	Mindanao PAIC	This study; Diesmos et al. 2015
Ranidae						
18	*Hylarana erythraea* (Schlegel, 1837) Green paddy frog	V	RF	LC	Throughout the Philippines (Introduced)	This study; Patindol 2016
19	*Hylarana grandocula* (Taylor, 1920)* Mindanao striped stream frog	I, II	RF	LC	Mindanao PAIC	This study; Obeña et al. 2021
20	*Sanguirana mearnsi* (Stejneger, 1905)* Mindanao torrent frog	-	-	LC	Mindanao PAIC	Diesmos et al. 2015
21	*Staurois natator* (Günther, 1858)* Mindanao rock frog	I, II	RF	LC	Mindanao PAIC	This study; Inger 1954
Rhacophoridae						
22	*Kurixalus appendiculatus* (Günther, 1858) Frilled tree frog	-	-	LC	Throughout the Philippines (Non-endemic)	Diesmos et al. 2015
23	*Leptomantis bimaculatus* Peters, 1867* White-spotted flying frog	II	RF	LC	Luzon & Mindanao PAICs	This study; Diesmos et al. 2015
24	*Nyctixalus spinosus* (Taylor, 1920)* Spiny cinnamon frog	I	FL	LC	Mindanao PAIC	This study; Diesmos et al. 2015
25	*Philautus leitensis* (Boulenger, 1897)* Mindanao bush frog	I	RF, FL	LC	Mindanao PAIC	This study; Gaulke 1994
26	*Philautus surdus* (Peters, 1863)* Philippine bush frog	I	FL	LC	Throughout the Philippines except Palawan PAIC (Endemic)	This study; Patindol 2016
27	*Philautus worcesteri* (Stejneger, 1905)* Worcester’s bush frog	II	SG	LC	Mindanao PAIC	This study
28	*Polypedates leucomystax* (Gravenhorst, 1829) Asiatic tree frog	III	OS, HH	LC	Throughout the Philippines (Non-endemic)	This study; Inger 1954
29	*Rhacophorus pardalis* Günther, 1858 Emerald flying frog	I, II	FL	LC	Throughout the Philippines (Non-endemic)	This study; Diesmos et al. 2015
REPTILIA (Order Squamata; Suborder Sauria)						
Agamidae						
30	*Bronchocela cristatella* (Kuhl, 1820) Green crested lizard	I	OS, FL	LC	Throughout the Philippines (Non-endemic)	This study; Sy 2023
31	*Draco bimaculatus* Günther, 1864* Black-spotted flying lizard	I, II	AP	LC	Mindanao PAIC	This study; Gaulke 1994
32	*Draco mindanensis* Stejneger, 1908* Giant Philippine flying lizard	II	SG	NT	Mindanao PAIC	This study; Gaulke 1994
33	*Draco ornatus* (Gray, 1845)* Ornate flying lizard	I	AP	LC	Mindanao PAIC	This study; Gaulke 1994
34	*Draco reticulatus* Günther, 1864* Reticulated flying lizard	-	-	LC	Mindanao PAIC (Samar & Leyte)	McGuire and Alcala 2000
35	*Gonocephalus semperi* (Peters, 1867)* Semper’s forest dragon	I	FL	LC	Throughout the Philippines (Endemic)	This study; Gaulke 1994
36	*Hydrosaurus pustulatus* (Eschscholtz, 1829)* Philippine sailfin lizard	II, V	RF	VU	Throughout the Philippines (Endemic)	This study; Gaulke 1994
Gekkonidae						
37	*Cyrtodactylus philippinicus* (Steindachner, 1867)* Philippine bent-toed gecko	-	-	LC	Throughout the Philippines (Endemic)	Gaulke 1994
38	*Cyrtodactylus sumuroi* Welton, Siler, Linkem, Diesmos, & Brown, 2010* Samar bent-toed gecko	I, II	FL	LC	Mindanao PAIC (Samar)	This study; Welton et al. 2010
39	*Gehyra mutilata* (Wiegmann, 1834) Four-clawed gecko	I	B	LC	Throughout the Philippines (Non-endemic)	This study; Gaulke 1994
40	*Gekko gecko* (Linnaeus, 1758) Tokay gecko	I	B	LC	Throughout the Philippines (Non-endemic)	This study; Patindol 2016
41	*Hemidactylus frenatus* Duméril & Bibron, 1836 Common house gecko	I	B, OS	LC	Throughout the Philippines (Non-endemic)	This study
42	*Hemidactylus platyurus* (Schneider, 1797) Flat-tailed house gecko	I	B	LC	Throughout the Philippines (Non-endemic)	This study; Gaulke 1994
43	*Hemiphyllodactylus typus* Bleeker, 1860 Indo-Pacific slender gecko	V	RF	LC	Throughout the Philippines (Non-endemic)	This study; Gaulke 1994
44	*Lepidodactylus aureolineatus* Taylor, 1915* Golden scaly-toed gecko	-	-	LC	Throughout the Philippines (Endemic)	Gaulke 1994
45	*Lepidodactylus planicaudus* Stejneger, 1905* Broad-tailed scaly-toed gecko	I	FL	LC	Throughout the Philippines (Endemic)	This study; Gaulke 1994
46	*Pseudogekko brevipes* (Boettger, 1897)* Orange-spotted leaf gecko	-	-	VU	Mindanao PAIC (Samar & Leyte)	Gaulke 1994
47	*Pseudogekko ditoy* Siler, Welton, Davis, Watters, Davey, Diesmos, Diesmos, & Brown, 2014* Dwarf leaf gecko	I	FL	DD	Mindanao PAIC (Samar & Leyte)	This study; Davis et al. 2015
48	*Pseudogekko pungkaypinit* Siler, Welton, Davis, Watters, Davey, Diesmos, Diesmos, & Brown, 2014* Striped leaf gecko	I	FL	LC	Mindanao PAIC	This study; Siler et al. 2014
Scincidae						
49	*Brachymeles orientalis* Brown & Rabor, 1967* Two-toned earth skink	I	FL	LC	Mindanao PAIC	This study; Obeña et al. 2021
50	*Brachymeles samad* Siler, Jones, Diesmos, Diesmos, & Brown, 2012* Waray earth skink	I, III	FL	LC	Mindanao PAIC (Samar & Leyte)	This study; Siler et al. 2012
51	*Brachymeles samarensis* Brown, 1956* Samar earth skink	-	-	NT	Mindanao PAIC (Samar)	Brown 1956
52	*Emoia atrocostata* (Lesson, 1829) Mangrove skink	-	-	LC	Throughout the Philippines (Non-endemic)	Brown and Alcala 1980
53	*Eutropis islamaliit* Barley, Diesmos, Siler, Martinez, & Brown, 2020* Striking Philippine sun skink	-	-	DD	Lubang, Semirara, & Samar	Barley et al. 2020
54	*Eutropis lapulapu* Barley, Diesmos, Siler, Martinez, & Brown, 2020* Lapu-Lapu’s sun skink	II	B, AP	LC	Throughout the Philippines (Endemic)	This study; Barley et al. 2020
55	*Eutropis multicarinata* (Gray, 1845)* Many-keeled sun skink	-	-	LC	Mindanao PAIC	Gaulke 1994; Barley et al. 2020
56	*Eutropis multifasciata* (Kuhl, 1820) Striped sun skink	I, II, V	B, OS, AP	LC	Throughout the Philippines (Non-endemic)	This study; Patindol 2016
57	*Lamprolepis smaragdina* (Lesson, 1829) Emerald tree skink	I	B, AP	LC	Throughout the Philippines (Non-endemic)	This study; Gaulke 1994
58	*Lipinia pulchella pulchella* (Gray, 1845)* Yellow-striped slender tree skink	I, II	AP, RF	LC	Throughout the Philippines except Palawan PAIC (Endemic)	This study; Gaulke 1994
59	*Lipinia quadrivittata* (Peters, 1867) Black-striped slender tree skink	I	AP	LC	Throughout the Philippines except Luzon PAIC (Non-endemic)	This study; Sy 2023
60	*Otosaurus cumingii* Gray, 1845* Philippine giant forest skink	II	FL	LC	Throughout the Philippines (Endemic)	This study; Patindol 2016
61	*Parvoscincus steerei* (Stejneger, 1908)* Steere’s pygmy forest skink	I	FL	LC	Throughout the Philippines except Palawan (Endemic)	This study; Gaulke 1994
62	*Pinoyscincus coxi coxi* (Taylor, 1915)* Cox’s Philippine skink	II	RF	LC	Mindanao PAIC	This study; Brown and Alcala 1980
63	*Pinoyscincus jagori jagori* (Peters, 1864)* Jagor’s Philippine skink	I, II	FL	LC	Mindanao PAIC	This study; Brown and Alcala 1980
64	*Pinoyscincus llanosi* (Taylor, 1919)* Father Llanos’ Philippine skink	I, II	FL, RF	NT	Mindanao PAIC (Samar & Leyte)	This study; Taylor 1919
65	*Pinoyscincus mindanensis* (Taylor, 1915)* Taylor’s Philippine skink	I	FL	LC	Mindanao PAIC	This study; Linkem et al. 2011
66	*Sphenomorphus acutus* (Peters, 1864)* Sharp-snouted forest skink	-	-	LC	Mindanao PAIC	Brown and Alcala 1980
67	*Sphenomorphus fasciatus* (Gray, 1845) Banded forest skink	I	AP	LC	Mindanao & Negros-Panay PAICs	This study; Brown and Alcala 1980
68	*Sphenomorphus variegatus* (Peters, 1867)* Black-spotted forest skink	-	-	LC	Mindanao PAIC	Gaulke 1994
69	*Tropidophorus grayi* Günther, 1861* Philippine keeled water skink	II	RF	LC	Luzon, Negros-Panay, & Mindanao PAICs	This study; Patindol 2016
Varanidae						
70	*Varanus samarensis* Koch, Gaulke, & Böhme, 2010* Waray water monitor	I, II, V	AP, FL, RF	LC	Mindanao PAIC (Samar & Leyte)	This study; Gaulke 1994
REPTILIA (Order Squamata; Suborder Serpentes)						
Colubridae						
71	*Ahaetulla prasina preocularis* (Taylor, 1822)* Philippine vine snake	I	FL	LC	Throughout the Philippines except Palawan & Sulu PAICs (Endemic)	This study; Leviton 1963
72	*Boiga angulata* (Peters, 1861)* Philippine blunt-headed cat snake	II	FL	LC	Throughout the Philippines except Palawan (Endemic)	This study; Gaulke 1994
73	*Boiga cynodon* (Boie, 1827) Dog-toothed cat snake	I, II	FL, OS	LC	Throughout the Philippines (Non-endemic)	This study
74	*Boiga dendrophila latifasciata* (Boulenger, 1896)* Mindanao mangrove cat snake	II	RF	LC	Mindanao PAIC	This study; Leviton 1963
75	*Calamaria lumbricoidea* Boie, 1827 Variable reed snake	I	B	LC	Mindanao PAIC	This study; Leviton 1963
76	*Chrysopelea paradisi variabilis* Mertens, 1968* Paradise tree snake	I	AP	LC	Throughout the Philippines (Endemic)	This study; Leviton 1963
77	*Coelognathus erythrurus erythrurus* (Duméril, Bibron, & Duméril, 1854)* Southern Philippine rat snake	-	-	LC	Mindanao PAIC	Leviton 1963
78	*Dendrelaphis marenae* Vogel & van Rooijen, 2008 Gaulke’s bronze-back tree snake	I	FL	LC	Throughout the Philippines (Non-endemic)	This study; Leviton 1963
79	*Dendrelaphis philippinensis* (Günther, 1879)* Philippine bronze-back tree snake	I, II	FL	LC	Throughout the Philippines except Palawan (Endemic)	This study; Leviton 1963
80	*Dryophiops philippina* Boulenger, 1896* Philippine keeled-bellied whip snake	-	-	DD	Luzon, Mindoro, & Negros-Panay PAICs	Gaulke 1994
81	*Lycodon capucinus* Boie, 1827 Common Asian wolf snake	-	-	LC	Throughout the Philippines (Introduced)	Leviton 1963
82	*Lycodon dumerilii* (Boulenger, 1893)* Dumeril’s Asian wolf snake	I	FL	LC	Mindanao PAIC	This study; Gaulke 1994
83	*Lycodon ferroni* Lanza, 1999* Ferron’s Asian wolf snake	-	-	VU	Mindanao PAIC (Samar)	Lanza 1999
84	*Rhabdophis auriculatus auriculatus* (Günther, 1858)* Günther’s white-lined water snake	I	FL	LC	Mindanao PAIC	This study; Leviton 1963
85	*Rhabdophis lineatus* (Peters, 1861)* Zigzag-lined water snake	II	RF	LC	Mindanao PAIC	This study; Leviton 1963
86	*Stegonotus muelleri* Duméril, Bibron, & Duméril, 1854* Müller’s rat snake	I, II	FL	NT	Mindanao PAIC	This study; Leviton 1963
87	*Tropidonophis dendrophiops* (Günther, 1883)* Spotted water snake	I	FL	LC	Mindanao PAIC	This study; Gaulke 1994
Cyclocoridae						
88	*Cyclocorus nuchalis taylori* Leviton, 1967* Taylor’s southern triangle-spotted snake	-	-	LC	Mindanao PAIC	Leviton 1963
89	*Levitonius mirus* Weinell, Paluh, Siler, & Brown, 2020* Waray dwarf burrowing snake	II	SG	DD	Mindanao PAIC (Samar & Leyte)	This study; Weinell et al. 2020
90	*Oxyrhabdium modestum* (Duméril, 1853)* Philippine shrub snake	I, II	RF	LC	Mindanao PAIC	This study; Leviton 1963
Elapidae						
91	*Calliophis philippinus* (Günther, 1864)* Philippine coral snake	-	-	-	Mindanao PAIC	Leviton 1963
92	*Naja samarensis* Peters, 1861* Samar cobra	LA	RF	LC	Mindanao PAIC	This study; Leviton 1963
93	*Ophiophagus bungarus* Schlegel, 1837 Sunda king cobra	-	-	-	Throughout the Philippines except Luzon PAIC (Non-endemic)	Patindol 2016
Pareidae						
94	*Aplopeltura boa* (Boie, 1828) Blunthead slug snake	I	FL	LC	Throughout the Philippines (Non-endemic)	This study; Villanueva et al. 2021
Psammodynastidae						
95	*Psammodynastes pulverulentus* (Boie, 1827) Common mock viper	I, II	RF, FL	LC	Throughout the Philippines (Non-endemic)	This study; Leviton 1963
Pythonidae						
96	*Malayopython reticulatus* (Schneider, 1801) Reticulated python	LA	RF, FL	LC	Throughout the Philippines (Non-endemic)	This study; Leviton 1963
Typhlopidae						
97	*Indotyphlops braminus* (Daudin, 1803) Brahminy blind snake	-	-	LC	Throughout the Philippines (Introduced)	Leviton et al. 2018
98	*Malayotyphlops ruber* (Boettger, 1897)* Samar blind snake	-	-	LC	Mindanao PAIC (Samar)	Boettger 1897
99	*Ramphotyphlops marxi* (Wallach, 1993)* Marx’s worm snake	I	FL	DD	Mindanao PAIC (Samar)	This study; Wallach 1993
100	*Ramphotyphlops olivaceus* (Gray, 1845) Olive-colored blind snake	-	-	LC	Sulu & Mindanao PAICs	Gaulke 1994
Viperidae						
101	*Trimeresurus flavomaculatus* (Gray, 1842)* Philippine pit viper	-	-	LC	Throughout the Philippines except Palawan (Endemic)	Leviton et al. 2018
102	*Tropidolaemus philippensis* (Gray, 1842)* Philippine keeled pit viper	I, II	RF	LC	Mindanao PAIC	This study; Leviton 1963
103	*Tropidolaemus subannulatus* (Gray, 1842) Bornean keeled pit viper	I, II	FL, RF	LC	Throughout the Philippines (Non-endemic)	This study; Gaulke 1994
REPTILIA (Order Testudines)						
Bataguridae						
104	*Cuora philippinensis* Blanck, Gaillard, Protiva, Wheatley, Shi, Liu, Ray, & Anders, 2023* Philippine box turtle	II	RF	-	Throughout the Philippines (Endemic)	This study; Gaulke 1994

### Herpetofaunal diversity of Samar Island Natural Park and Samar Island, Philippines

This paper presents a comprehensive summary of the herpetofauna documented within the Samar Island Natural Park and, more broadly, across Samar Island. Our surveys within the SINP recorded a total of 79 species of amphibians and reptiles. This number represents an increase of 39 species compared to previously published accounts from SINP (Patindol 2016; Villanueva et al. 2021), elevating the total recorded species within the natural park to 85. Furthermore, our findings include at least three species—*Leptobrachium lumadorum*, *Philautus worcesteri*, and *Boiga cynodon*—new to the known herpetofauna of Samar Island, thereby increasing the island’s total documented reptile and amphibian species to 104 (Table 1). The 85 species identified within the SINP represent approximately 81% (85 out of 104 species) of the total herpetofauna currently known from Samar Island.

In compiling our comprehensive checklist for Samar Island, certain historical records were excluded due to potential misidentifications or the use of outdated nomenclature. Excluded from our list are *Limnonectes woodworthi*, *L. visayanus*, *Platymantis dorsalis*, *Cyrtodactylus annulatus*, *Liopeltis philippinus* (a record also questioned by Gaulke 1994), *Hemibungarus calligaster*, and *Oligodon ancorus* (an unconfirmed listing according to Leviton 1963), all of which, as presently understood, are restricted to other PAICs. *Brachymeles gracilis* and *B. hilong* were also excluded, as the distribution of both are now restricted to Mindanao Island (Siler et al. 2012). Finally, *Polypedates hecticus*, a species purportedly endemic to Samar Island, has been synonymized with *Polypedates leucomystax* by Diesmos et al. (2015).

Our field surveys failed to find several species that were documented in earlier studies. Their apparent absence is likely attributable to limited detectability rather than actual absence from the study sites, particularly for arboreal and fossorial taxa. Notably, canopy-dwelling and arboreal species—including *Draco reticulatus*, *Lepidodactylus aureolineatus*, *Pseudogekko brevipes*, and *Dryophiops philippina*—were not detected, a pattern consistent with the known underrepresentation of such species in ground-based visual encounter surveys. Similarly, fossorial and litter-dwelling lizards such as *Brachymeles samarensis*, *Sphenomorphus acutus*, *S. variegatus*, and several typhlopid snakes are typically found only through intensive microhabitat searches and are therefore infrequently encountered.

Large-bodied or wide-ranging predators, including *Ophiophagus bungarus*, *Calliophis philippinus*, *Trimeresurus flavomaculatus*, and *Lycodon ferroni*, were likewise rarely observed, which is not unexpected given their inherently low encounter rates and extensive home ranges. In addition, some historically recorded species are closely associated with habitat types that were poorly represented or not sampled during our surveys, such as beach forests or mangroves (*Emoia atrocostata*, *Fejervarya moodiei*). Consequently, the non-detection of these species should not be interpreted as evidence of local extirpation but rather as an artefact of sampling. Nevertheless, for a small number of Samar-endemic taxa with limited historical records, ongoing habitat modification combined with the absence of recent observations underscores the need for targeted surveys to better assess their current conservation status.

### Comments on particular species

The species discussed below represent taxa of biogeographic and conservation significance to Samar Island, including single-island endemics, Samar–Leyte endemics, newly documented island records, rediscoveries of historically known taxa, species with problematic taxonomic histories, and putatively undescribed species. Several of these taxa also illustrate patterns of pronounced micro-endemism, a characteristic increasingly recognized in the herpetofauna of Samar, particularly among lowland and limestone-associated species. This type of micro-endemism likely reflects a combination of isolation, habitat specialization, and limited dispersal across karst landscapes and riverine barriers.

#### Class *Amphibia*

**Family Ceratobatrachidae**

##### *Platymantis bayani* Siler, Alcala, Diesmos, & Brown, 2009

Fig. 3B

**Material examined**. Philippines • 2 ♀, 1 ♂; Province of Samar, Municipality of Paranas, Barangay Tenani, Samar Island Natural Park; 11.81405°N, 125.16095°E; 200 m a.s.l.; 24 October 2021; Y.L. Del Prado, M.L. Diesmos, P.M. Kim, and J. Fernandez leg.; Ground; UST-HRC 837–838, 843 • 4 ♀, Municipality of Calbiga, Barangay Panayuran, Langun-Gobingob Cave; 11.81821°N, 125.19424°E; 204–244 m elevation; P.M. Kim; N.A. Caguimbal; J. Fernandez leg.; Limestone crevices; UST-HRC 1008–1010, UST-HRC 1016.

**Figure 3.**
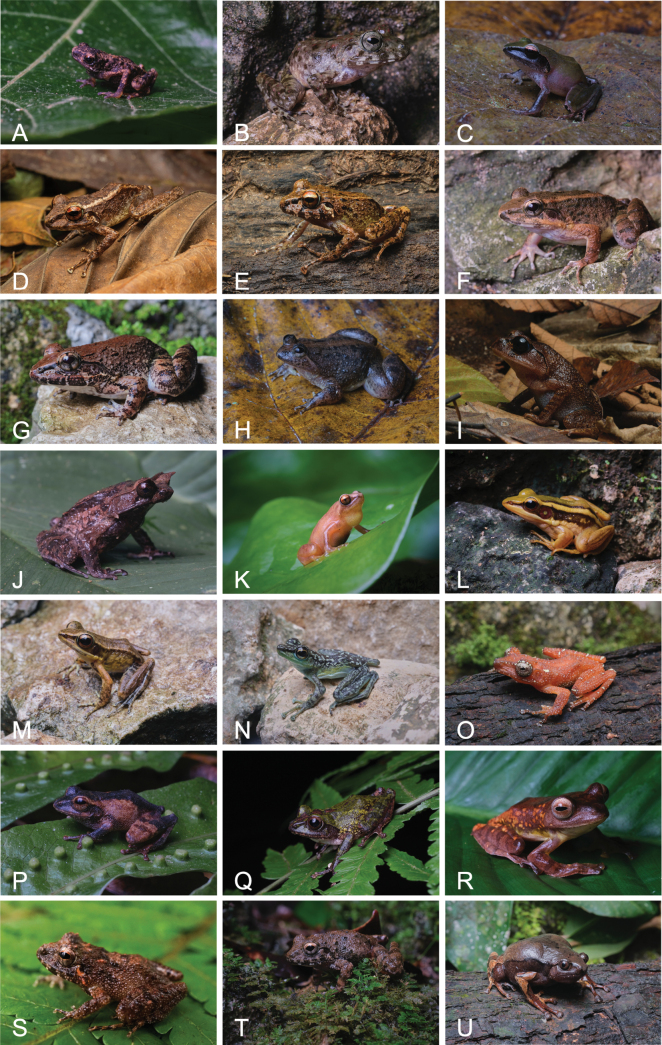
Amphibian species documented within SINP: **A**. *Pelophryne lighti*; **B**. *Platymantis bayani*; **C**. *P. corrugatus*; **D**. *P. guentheri*; **E**. *P. rabori*; **F**. *Limnonectes leitensis*; **G**. *L. magnus*; **H**. *Occidozyga laevis*; **I**. *Leptobrachium lumadorum*; **J**. *Pelobatrachus stejnegeri*; **K**. *Oreophryne anulata*; **L**. *Hylarana erythraea*; **M**. *H. grandocula*; **N**. *Staurois natator*; **O**. *Nyctixalus spinosus*; **P**. *Philautus leitensis*; **Q**. *P. worcesteri*; **R**. *Rhacophorus pardalis*; **S**. *Platymantis* sp. 1; **T**. *Platymantis* sp. 2; **U**. *Kaloula* sp.

**Identification**. Large body size (SVL: 37–51 mm); large eye diameter (ED: 5.44–5.65 mm); disks of digits widely expanded, larger in fingers than in toes; presence of light pink dorsal dermal tubercles; completely visible tympanum; absence of dorsolateral stripes; terrestrial microhabitat in karst forest (Siler et al. 2009).

**Remarks**. The species, since its description in 2009, is still known only from Samar Island. All specimens we encountered were limited to limestone boulders and outcrops, either on the ground or inside karst crevices. Despite the rains, no advertisement calls of *P. bayani* were heard and recordings are still non-existent.

##### *Platymantis* sp. 1

Fig. 3S

**Material examined**. Philippines • 1 ♀, 1 ♂; Province of Samar, Municipality of Paranas, Barangay San Isidro, Liao Cave; 11.8587°N, 125.21109°E; 49–117 m a.s.l.; 02 November 2021; Y.L. Del Prado, P.M. Kim, R.L. Venturina, N.A. Caguimbal, J. Fernandez, and A.C. Diesmos leg.; Ground; UST-HRC 977–978.

**Remarks**. We collected two individuals of an acoustically distinctive terrestrial species of *Platymantis* along the ridges of the Ulot Watershed. The species was not present in other sites. It is small in size (SVL: ♂ 18 mm and ♀ 20 mm).

##### *Platymantis* sp. 2

Fig. 3T

**Material examined**. Philippines • 5 ♀, 4 ♂; Province of Samar, Municipality of Paranas, Barangay Tenani, Samar Island Natural Park; 11.81405°N, 125.16095°E; 200 m a.s.l.; 30 October 2021; Y.L. Del Prado, M.L. Diesmos, P.M. Kim, and J. Fernandez leg.; Ground; UST-HRC 839, UST-HRC 846, UST-HRC 873–874, UST-HRC 879, UST-HRC 920–922, UST-HRC 931, UST-HRC 953 • 2 ♂; Barangay San Isidro, Liao Cave; 11.8587°N, 125.21109°E; 49–117 m a.s.l.; 08 November 2021; Y.L. Del Prado, M.L. Diesmos, P.M. Kim, and J. Fernandez leg.; Ground; UST-HRC 1012–1013.

**Remarks**. Several individuals of this species of *Platymantis* were recorded during our surveys. This taxon is distinguished by a unique advertisement call characterized by a long, continuous series of clicks. The species is moderate in size (SVL: ♂ 26–31 mm, ♀ 27–35 mm) and was relatively common in the karst environments of SINP and Liao Cave.

#### Family Megophryidae

***Leptobrachium lumadorum* Brown, Siler, Diesmos, & Alcala, 2010**

Fig. 3I

**Material examined**. Philippines • Province of Samar, Municipality of Paranas, Barangay San Isidro, Ulot Watershed; 11.8587°N, 125.21109°E; 49–117 m a.s.l.; 13 March 2016; A.C. Diesmos obs.

**Identification**. Head large; eyes large; tips of digits blunt, rounded; dorsal coloration dark brown; presence of blue scleral arc under eyelids; presence of broad canthal stripe.

**Remarks**. The endemic species is known to be broadly distributed across Mindanao and Basilan Islands, occupying a range of habitat types (Brown et al. 2009). During our surveys in SINP, we detected and recorded the species’ distinctive low-frequency advertisement calls, representing the first confirmed distribution record for *L. lumadorum* on Samar Island. Given this occurrence on the northeastern margin of the Mindanao PAIC and the species’ broad ecological tolerance, it is plausible that *L. lumadorum* also occurs on other islands within the region.

#### Family Microhylidae

***Kaloula* sp**.

Fig. 3U

**Material examined**. Philippines • 1 ♀; Province of Samar, Municipality of Paranas, Barangay Tenani, Samar Island Natural Park; 11.81405°N, 125.16095°E; 200 m a.s.l.; 30 October 2021; Y.L. Del Prado, P.M. Kim, J. Fernandez, R.L. Venturina, N.A. Caguimbal, and A.C. Diesmos leg.; Treehole; UST-HRC 931.

**Remarks**. A single specimen of a morphologically and acoustically distinctive *Kaloula* was collected from a tree hole within the limestone forest habitat of SINP. We tentatively assign this individual to the undescribed species of *Kaloula* previously reported from Leyte, Samar (Blackburn et al. 2013), and northeastern Mindanao (Sanguila et al. 2016). SVL: 40 mm.

#### Family Rhacophoridae

***Philautus worcesteri* (Stejneger, 1905)**

Fig. 3Q

**Material examined**. Philippines • Province of Samar, Municipality of Paranas, Barangay San Isidro, Ulot Watershed; 11.8587°N, 125.21109°E; 49–117 m a.s.l.; 13 March 2016; A.C. Diesmos obs.

**Identification**. Dorsum smooth; tips of digits enlarged, rounded; dorsal coloration reddish brown with green blotches; ventral white

**Remarks**. This larger species of *Philautus* was previously documented exclusively from Mindanao Island, where it is known to be widely distributed (Brown and Alcala 1994; Sanguila et al. 2016). During our field surveys within SINP, we detected and recorded the species’ distinctive vocalizations in the forested areas of the Ulot Watershed. These observations constitute the first confirmed records of the species occurring outside of Mindanao.

#### Class Reptilia

**Family Agamidae**

##### *Bronchocela cristatella* (Kuhl, 1820)

Fig. 4A

**Material examined**. Philippines • 2 ♀, 1 ♂; Province of Samar, Municipality of Paranas, Barangay Tenani, Samar Island Natural Park; 11.81405°N, 125.16095°E; 200 m a.s.l.; 30 October 2021; Y.L. Del Prado, M.L. Diesmos, P.M. Kim, and J. Fernandez leg.; On fern; UST-HRC 860–862.

**Figure 4.**
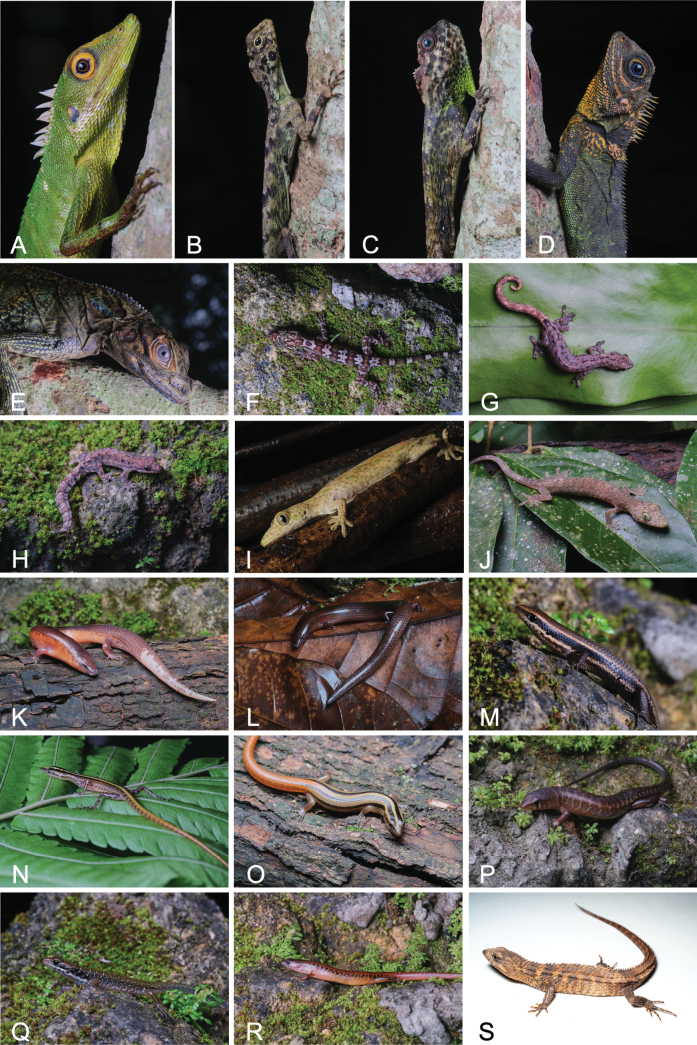
Photographs of lizard species documented within SINP: **A**. *Bronchocela cristatella*; **B**. *Draco bimaculatus*; **C**. *D. ornatus*; **D**. *Gonocephalus semperi*; **E**. *Hydrosaurus pustulatus*; **F**. *Cyrtodactylus sumuroi*; **G**. *Hemiphyllodactylus typus*; **H**. *Lepidodactylus planicauda*; **I**. *Pseudogekko ditoy*; **J**. *P. pungkaypinit*; **K**. *Brachymeles orientalis*; **L**. *B. samad*; **M**. *Eutropis lapulapu*; **N**. *Lipinia pulchella*; **O**. *L. quadrivittata*; **P**. *Pinoyscincus jagori*; **Q**. *P. llanosi*; **R**. *P. mindanensis*; **S**. *Tropidophorus grayi*.

**Identification**. Adult SVL: 80–90 mm; Tail Length: 265–300 mm; pointed snout; presence of white nuchal crest; dorsal crest much shorter and with uniform green dorsal coloration; from congeners, shorter nuchal crest; four to ten upper scale rows pointing upwards; 10–12 ventral scale rows; absence of labial stripe.

**Remarks**. As currently understood, *B. cristatella* is widespread throughout Southeast Asia including the Philippines (Hallermann 2005). However, Sanguila et al. (2016) suspect that Philippine *B. cristatella* are likely different from the type locality, suggesting a taxonomic review using both molecular and morphological data. Despite the possibly problematic taxonomy for the group, we chose to follow Hallermann (2005) pending taxonomic resolution for Philippine populations.

##### *Gonocephalus semperi* (Peters, 1867)

Fig. 4D

**Material examined**. Philippines • 2 ♀, 2 ♂; Province of Samar, Municipality of Paranas, Barangay Tenani, Samar Island Natural Park; 11.81405°N, 125.16095°E; 200 m a.s.l.; 23 October 2021; Y.L. Del Prado, M.L. Diesmos, P.M. Kim, and J. Fernandez leg.; On saplings; UST-HRC 841, UST-HRC 859, UST-HRC 878, UST-HRC 919.

**Identification**. Adult SVL: 90–100 mm; in males, Nuchal Crest: lanceolate, not continuous with smaller dorsal crest; Supralabials: ten or eleven; Infralabials: nine or ten; Ventral Scales: keeled; Gular Sac of males: orange-yellow marbled with green; dorsal coloration black or dark brown mixed with green; tail with broad dark brown annuli.

**Remarks**. The taxonomy of Philippine *Gonocephalus* is still unresolved. As currently understood, all three species are overlapping in distribution where type localities of all three are given as “Philippines” with no specific localities (Gray 1845; Peters 1867; Boulenger 1885). In the most recent phylogenetic analyses of the group, Welton et al. (2017) designated Samar-Leyte populations as *Gonocephalus semperi* which we chose to follow here.

#### Family Gekkonidae

***Pseudogekko ditoy* Siler, Welton, Davis, Watters, Davey, Diesmos, Diesmos, & Brown, 2014**

Fig. 4I

**Material examined**. Philippines • 1 ♀; Province of Samar, Municipality of Paranas, Barangay Tenani, Samar Island Natural Park; 11.81405°N, 125.16095°E; 200 m a.s.l.; 11 November 2021; P.M. Kim, N.A. Caguimbal, and J. Fernandez leg.; Along road; UST-HRC 1017.

**Identification**. Small body size, SVL: 56 mm; Supralabials: 16; Infralabials: 17; Circumorbitals: 44; Finger III Scansors: 14; Toe IV Scansors: 18; Ventral Scale Count: 119; ciliary ring color undifferentiated.

**Remarks**. We identify the lone specimen as *P. ditoy* based on morphometrics, meristics, and species range. We also report the presence of precloacal pores on the gravid female specimen, an observation initially described by Davis et al. (2015). However, we note the presence of faint neon green spotting on head, dorsolaterals, and limbs, and the presence of tail bands which are supposedly diagnostic of *P. chavacano* of Western Mindanao (Siler et al. 2014).

#### Family Colubridae

***Boiga cynodon* (Boie, 1827)**

Fig. 5B

**Material examined**. Philippines • 1 ♀, 1 ♂; Province of Samar, Municipality of Paranas, Barangay Tenani, Samar Island Natural Park; 11.81405°N, 125.16095°E; 200 m a.s.l.; 30 October 2021; Y.L. Del Prado, M.L. Diesmos, P.M. Kim, N.A.L. Caguimbal, R.L. Venturina, J. Fernandez, and A.C. Diesmos leg.; Understory; UST-HRC 892, UST-HRC 936 • 1 sex indet.; Barangay San Isidro, Ulot Watershed; 11.8587°N, 125.21109°E; 49–117 m a.s.l.; 02 November 2021; P.M. Kim, N.A.L. Caguimbal, and J. Fernandez leg.; Understory; UST-HRC 991.

**Figure 5.**
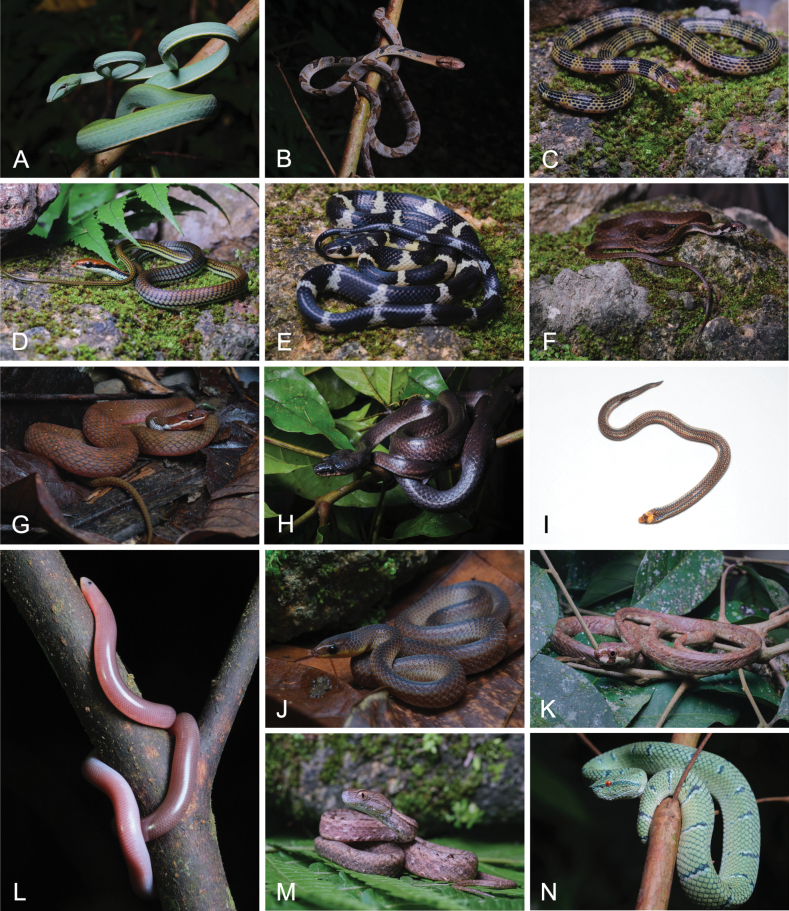
Photographs of snake species documented within SINP: **A**. *Ahaetulla prasina preocularis*; **B**. *Boiga cynodon*; **C**. *Calamaria lumbricoidea*; **D**. *Dendrelaphis philippinensis*; **E**. *Lycodon dumerilii*; **F**. *Rhabdophis auriculatus auriculatus*; **G**. *R. lineatus*; **H**. *Stegonotus muelleri*; **I**. *Levitonius mirus*; **J**. *Oxyrhabdium modestum*; **K**. *Aplopeltura boa*; **L**. *Ramphotyphlops marxi*; **M**. *Psammodynastes pulverulentus*; **N**. *Tropidolaemus subannulatus*.

**Identification**. Head large, distinct from neck; eyes large; large adult size (SVL: 986–1025 mm); brown to dark brown dorsal coloration; venter cream; distinct black stripe from eye to angle of jaw; presence of dark bands on body; Midbody Dorsal Scale Row: 23; Infralabials: 13, eight anterior to center of eye.

**Remarks**. During our 2021 field surveys, three individuals of *Boiga cynodon*, a species widely distributed throughout Southeast Asia (Uetz et al. 2025), were collected. Although the species is known to occur broadly within the Philippines (Leviton et al. 2018) and has been previously documented on the adjacent island of Leyte (Denzer et al. 1994), these specimens represent the first records of *B. cynodon* from Samar Island.

#### Family Typhlopidae

***Ramphotyphlops marxi* (Wallach, 1993)**

Fig. 5L

**Material examined**. Philippines • 1 sex indet.; Province of Samar, Municipality of Paranas, Barangay Tenani, Samar Island Natural Park; 11.81405°N, 125.16095°E; 200 m a.s.l.; 23 October 2021; Y.L. Del Prado, M.L. Diesmos, P.M. Kim, and J. Fernandez leg.; Understory; UST-HRC 817 • 1 ♀; Barangay San Isidro, Ulot River; 11.8587°N, 125.21109°E; 49–117 m a.s.l.; 05 November 2021; P.M. Kim, N.A.L. Caguimbal, and J. Fernandez leg.; Understory; UST-HRC 1002.

**Identification**. SVL: 160 and 264 mm; Rostral Scale: broad, parallel-sided, and beaked; Midbody Scale Rows: 30; ocular scale overlapping the third supralabial; tail relatively long (5% and 7.5% of total length), pointed; above coloration pinkish; below white.

**Remarks**. Our field investigations led to the significant rediscovery of *Ramphotyphlops marxi*, a blind snake species previously known only from two historical specimens. The species was originally described by Wallach (1993) based on a single specimen (FMNH 96520) collected in 1957 from Samar Island by D.S. Rabor. Wallach and Gemel (2019) subsequently identified an additional specimen (NMW 40433) within the collections of the Naturhistorisches Museum Wien (NMW), which lacked specific collection data. The two specimens collected during our recent surveys represent the third and fourth vouchered records for this species.

The paucity of information regarding *R. marxi* is largely attributable to the historical lack of intensive field surveys in the region, potentially compounded by the species’ inherent rarity, restricted range, and cryptic habits. Both individuals were collected on saplings within the limestone forests of SINP, suggesting a potentially arboreal lifestyle for this blind snake species and a possible restriction to limestone karst habitats. *Ramphotyphlops marxi* is currently listed as Data Deficient by the IUCN (Kim 2022), underscoring the critical importance of protecting remaining natural habitats on Samar Island to ensure the conservation of this poorly understood species.

### Biogeographic affinities and endemism

Species endemism of Samar herpetofauna is remarkably high with 72% (75 of 104 species) being restricted to the Philippines. Of these Philippine endemics, nearly half (37 of 75 species) are found exclusively within the Mindanao Biogeographic Region. This strong affinity with the southern Philippines aligns with the Pleistocene Aggregate Island Complex (PAIC) Diversification Model, which explains that repeated cycles of sea-level lowering during the Pleistocene intermittently connected major islands, facilitating dispersal (Inger 1954; Heaney 1985; Brown and Diesmos 2001; Brown et al. 2013). However, while the PAIC system explains broad patterns of faunal similarity and regional endemism, it does not fully account for the substantial within-island diversification observed in the eastern Visayas region.

The presence of eight Samar-Leyte endemic species and six Samar-endemic species reaffirms that the herpetofauna of Samar is not merely a subset of that of Mindanao (Welton et al. 2010; Siler et al. 2012; Diesmos et al. 2020; Weinell et al. 2020), suggesting more complex biogeographical patterns are at play. Although Samar and Leyte were connected to Mindanao during the Pleistocene, subsequent geographic isolation following sea-level rise likely promoted allopatric differentiation and the evolution of island-restricted endemic lineages.

Topographic complexity, habitat heterogeneity, and long-term isolation are some of the major drivers of within-island diversification (Brown and Diesmos 2009; Brown et al. 2013, 2015b; Siler et al. 2014). Fine-scale differentiation may be particularly important in taxa with specialized ecological requirements or limited dispersal abilities, such as many forest-dependent amphibians and reptiles. In such groups, isolation among river systems or distinct habitat types (e.g., limestone karst forests) may have allowed repeated and localized diversification within the same island (Welton et al. 2010; Brown et al. 2013, 2015b).

Importantly, land connections between Samar-Leyte and Mindanao may have been short-lived or ecologically fragmented, limiting sustained gene flow among populations. Temporal fluctuations in habitat availability likely produced repeated cycles of connectivity and isolation within islands, further facilitating intra-island diversification. The observation that ~ 38% (14 species) of the 37 Mindanao-PAIC–endemic species are restricted to the islands of Samar and Leyte underscores that these islands function not merely as peripheral components of the PAIC but as important, independent centers of evolutionary divergence.

Samar Island now harbors nearly 55% of the amphibian and reptilian species that are known from the Mindanao PAIC and ~ 21% of all species in the Philippines. This underscores that Samar represents a key center of diversity and endemism for Philippine herpetofauna. Taken together, the high levels of species richness and endemism and pronounced within-island diversification reinforce the island’s critical role as an important reservoir of Philippine biodiversity.

### Importance of limestone karst ecosystems

Limestone karsts are globally recognized as critical forest ecosystems for global biodiversity, harboring unique assemblages of flora and fauna specifically adapted to these harsh and distinct environments (Clements et al. 2006; Tolentino et al. 2020). They serve as biodiversity reservoirs (Schilthuizen 2004), supporting a high degree of species endemism due to their unique and specialized environmental conditions.

Our surveys in SINP underscore the immense ecological value of this protected habitat with at least 43 species of amphibians and reptiles thriving within the forests over limestone. Notably, the limestone forest frog *Platymantis bayani* was exclusively observed in karst habitats, confirming previous observations by Siler et al. (2009). Limestone forest habitats play an irreplaceable role in supporting the survival of this unique group of frogs.

The Philippines boasts of an extensive network of limestone karst landscapes, encompassing ca 35,000 km^2^ or nearly 12% of its total land area (Clements et al. 2006; Restificar et al. 2006; Brown et al. 2015a). While these unique geological formations are ecologically invaluable, they also represent a crucial resource for human communities, functioning as major groundwater reservoirs and regulating hydrological processes (Clements et al. 2006). Unfortunately, these vital ecosystems face severe and escalating threats. Mining and quarrying of limestone lead to direct habitat destruction and fragmentation. Conversion into agricultural lands further exacerbates the degradation of these fragile environments, resulting in habitat loss, altered hydrological cycles, and increased erosion. The continued degradation of forests over limestone poses a significant threat to the unique biodiversity they support and the essential ecosystem services they provide. Understanding the unique characteristics and critical threats to these karst ecosystems is paramount for developing effective conservation strategies.

## Conclusions

Our findings highlight the richness and high endemism of the herpetofauna of Samar Island, with significant levels of unrecognized biodiversity. The further discovery of potentially novel species on the island is not unexpected. Recent fieldwork have already found exceptional additions to the fauna, such as *Platymantis navjoti* and *Levitonius mirus* (Diesmos et al. 2020; Weinell et al. 2020). These discoveries of new species highlight considerable gaps in our current knowledge regarding the biodiversity of the Philippines. Continuous and intensive field research in the different habitats on Samar, including Leyte and adjacent smaller islands (e.g., Biliran, Biri, Buad, Daram, Maripipi), is crucial to fully document and understand the unique herpetological richness of this region.

## Additional information

### Use of AI

No use of AI was reported.

### Author ORCIDs

Mae Lowe L. Diesmos https://orcid.org/0009-0006-7876-9989

Yñigo Luis C. del Prado https://orcid.org/0009-0009-7766-4650

Russell Evan L. Venturina https://orcid.org/0009-0000-5625-8076

Arvin C. Diesmos https://orcid.org/0000-0001-7272-3927

### Data availability

All of the data that support the findings of this study are available in the main text.
